# The more smoking the more cataract: A study on smoking, snus use and cataract in a Swedish population

**DOI:** 10.1111/aos.16770

**Published:** 2024-10-18

**Authors:** Moa Nordström, Madeleine Zetterberg, Kjell Torén, Linus Schiöler, Mathias Holm

**Affiliations:** ^1^ Department of Clinical Neuroscience, Institute of Neuroscience and Physiology, Sahlgrenska Academy University of Gothenburg Gothenburg Sweden; ^2^ Department of Ophthalmology Sahlgrenska University Hospital, Region Västra Götaland Mölndal Sweden; ^3^ Occupational and Environmental Medicine, School of Public Health and Community Medicine, Institute of Medicine, Sahlgrenska Academy University of Gothenburg Gothenburg Sweden

**Keywords:** cataract, cataract surgery, moist smokeless tobacco, population‐based study, risk factors, smoking, snus

## Abstract

**Purpose:**

To examine the prevalence of self‐reported cataract and cataract surgery, and the incidence of cataract surgery, in relation to smoking and use of the moist smokeless tobacco product snus.

**Methods:**

In 2014/2015, individuals born in 1951 (*n* = 18 055) in the Västra Götaland County, Sweden, were invited to participate. Of these, 9743 (54%) accepted participation and 9316 (52%) remained after exclusion criteria were applied. Participants answered a questionnaire with items about eye conditions, smoking, snus, gender, education, asthma, chronic obstructive pulmonary disease, corticosteroid use, diabetes mellitus, weight and height. Prevalence ratios (PRs) for self‐reported cataract and cataract surgery were calculated. The incidence of cataract surgery was assessed, and hazard ratios (HRs) were presented.

**Results:**

Having ever smoked was associated with a higher prevalence ratio of self‐reported cataract (PR 1.19, 95% confidence interval [CI] 1.04–1.35) and cataract surgery (PR 1.27, 95% CI 1.06–1.53), compared to those who had never been daily smokers. Currently, a smoker was associated with a higher HR of cataract surgery (HR 1.34, 95% CI 1.04–1.74), as well as having been a former smoker (HR 1.27, 95% CI 1.03–1.56). Total years of smoking were associated with an increased risk for cataract surgery (HR 1.05, 95% CI 1.02–1.08 for 5 years of smoking). Snus use was not associated with an increased prevalence of cataract or incidence of cataract surgery, except among women who were current snus users (HR for cataract surgery 2.04, 95% CI 1.16–3.60 *n* = 108).

**Conclusion:**

Smoking is associated with a higher prevalence of cataracts, and a higher incidence of cataract surgery, indicating a dose–response relationship. However, there was no firm association between snus use and cataract.

## 1 INTRODUCTION

Age‐related cataract is the leading cause of blindness globally, with 17 million people worldwide reported to be blind due to cataract in 2020 (Bourne et al., 2021). The overall prevalence of cataract and cataract surgery in Sweden was 23.4% (27.2% in women and 19.1% in men) in 70‐year‐olds (Nordström et al., 2022), and the prevalence of cataract surgery has been estimated to 8% in 63–64‐year olds in 2022 in Sweden (Bro et al., 2024).

Several studies have shown a strong association between smoking and cataract, especially nuclear cataract (Kelly et al., 2005). There are fewer population‐based studies investigating the long‐term longitudinal relationship between smoking and cataract (Hiller et al., 1997; Klein et al., 2003; Leske et al., 1998; Lindblad et al., 2005; Tan et al., 2008), the effect of smoking cessation, and dose–response effect. Some reports indicate that the risk for cataract persists for a long period in those who have formerly smoked (Christen et al., 2000; Kelly et al., 2005; Lindblad et al., 2005; Weintraub et al., 2002). Oxidative stress is considered to be involved in the aetiology of age‐related cataract, especially nuclear (located in the center of the lens, the nucleus) and cortical (located in the cortex of the lens, encircling the nucleus) cataracts. As the lens ages, the lens nucleus becomes more susceptible to oxidation and the ability to repair oxidative damage decreases (Truscott, 2005). Smoking causes oxidative stress to the lens by generating free radicals and decreasing levels of antioxidants (Chow et al., 1986; Spector, 1995). This is believed to generate crosslinking between lens proteins, ultimately resulting in light‐scattering protein aggregates, especially in nuclear cataract, and also loss of lens fibre structure, leading to cortical cataract spokes (Al‐Ghoul et al., 1996; Brown et al., 1989).

Other risk factors for cataract induce oxidative stress, among them diabetes, and many studies show an association between diabetes and cataract (Klein et al., 1998; Rowe et al., 2000). Oral and inhaled corticosteroids are associated with cataract (Wang et al., 2009), and an association between asthma and cataract has been shown (Prokofyeva et al., 2013), which could be due to the use of corticosteroids.

A meta‐analysis, where participants with previous cataract surgery were excluded, demonstrated that myopia is associated with prevalent cataract, which could be due to a myopic shift in eyes with nuclear cataract, but an association with incident cataract could not be found (Pan et al., 2013). However, another study shows an association between myopia and posterior subcapsular cataract, and between myopia and incident cataract surgery (Younan et al., 2002). An association between BMI and age‐related cataract has been found in one study (Hiller et al., 1998) whilst another study found no association between BMI and early onset cataract (Zhang et al., 2021). Lower educational level, as a proxy indicator of socioeconomic status, is associated with cataract in several studies (Delcourt et al., 2000; Leske et al., 1997), which could be due to life‐style behaviour and health status.

In Sweden, like in many other high‐income countries, the prevalence of smoking decreases (Marcon et al., 2018). However, the use of snus, a moist smokeless tobacco product, has increased in Sweden in recent years, and the use of snus has recently become common elsewhere, for example in the United States (U.S.). An increased risk for diabetes type‐2 in snus users (as in smokers) is found in some studies, especially in those with high consumption (Carlsson et al., 2017). Other studies show associations between snus use in pregnancy and an increased risk for neonatal apnea, preterm birth, ‘small for gestational age’ births, and stillbirth (Brinchmann et al., 2023). There is also a large pooled analysis of eight prospective studies showing an association between snus use and increased all‐cause mortality among men (Byhamre et al., 2021). Users of snus are, just like smokers, exposed to high levels of nicotine and tobacco‐specific nitrosamines (Norweigan Institute of Public Health, 2019). In contrast, cadmium levels are elevated in smokers, but not in consumers of smokeless tobacco in the U.S. (Marano et al., 2012; Prasad et al., 2016). Cadmium has been found in higher concentrations in lenses with cataract than without cataract, and it has been argued that the accumulation of heavy metals like cadmium is involved in cataractogenesis (Cekic, 1998). Smoking in relation to cataract is relatively well studied, but to the best of our knowledge, the impact of snus use on cataract incidence has not been investigated, although an association between smokeless tobacco and cataract was shown in a cross‐sectional study (Raju et al., 2006). Studies that compare the effect of different types of tobacco use on cataract are scarce.

In this study, we examine the prevalence of cataract and cataract surgery, as well as the incidence of cataract surgery in relation to smoking and snus use in a large Swedish general population sample aged 63–64 years.

## 2 MATERIALS AND METHODS

**FIGURE 1:**
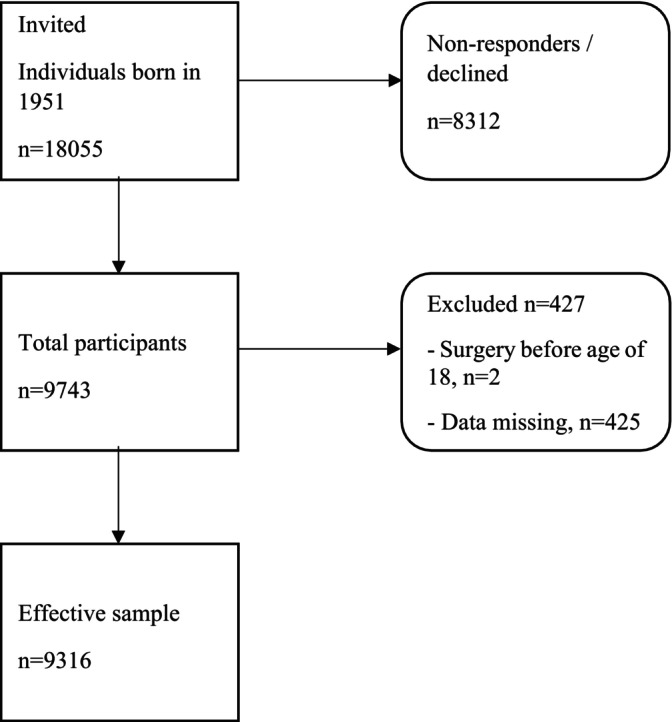
Flowchart showing participants born in 1951, living in Västra Götalands County, Sweden, who received a questionnaire about eye diseases and occupational exposures in 2014–2015. A number of 18 055 were invited; 8312 declined and 427 were excluded, which resulted in an effective sample of 9316 participants.

Cataract Western Sweden is a large questionnaire‐based cohort study. In 2014 and 2015, the ‘Cataract and working environment’ questionnaire was sent to all individuals in the Västra Götaland County born in 1951. Of 18 055 recipients, a total of 9747 (54%) answered the questionnaire. The questionnaire comprised items about cataract diagnosis, cataract surgery, heredity for cataract surgery, myopia, eye trauma, asthma, chronic obstructive pulmonary disease, diabetes mellitus, corticosteroid medication use, history of tobacco use, occupational exposures, gender, weight, height, and educational level. A prerequisite for study inclusion was that the participant answered the questions about cataract, gender, smoking, snus use, and education level, as did 9318 participants. If cataract surgery had been performed before the age of 18, the participant was excluded (*n* = 2). A flow chart of the inclusion process is shown in Figure 1. The study was approved by the Regional Ethical Review in Gothenburg, Gothenburg University Board (D‐nr 204‐14), and informed consent was obtained from all participants. The study was performed in accordance with the tenets of the Declaration of Helsinki.

In order to define the outcomes, the answers to the questions ‘Have you been told by an eye doctor or optician that you have cataract?’ and ‘Have you had cataract surgery?’ were used. Self‐reported cataract was defined as an affirmative answer to either of the questions and self‐reported cataract surgery as an affirmative answer to the latter question. The participants also had to state which year they were diagnosed with cataract or had cataract surgery. In a previous study, sensitivity for self‐reported cataract surgery was identified as 90.7%, specificity as 99.2%, positive predictive value (PPV) as 0.944, and negative predictive value (NPV) as 0.986 (Nordström et al., 2022). The sensitivity for self‐reported cataract diagnosis was identified as 52.9%, specificity as 92.3%, PPV as 0.730, and NPV as 0.832 (Nordström et al., 2022).

‘Having ever smoked daily’ was defined as a positive answer to ‘Have you ever smoked on a daily basis for at least a month?’. ‘Pack‐year’ was defined as the number of packs of cigarettes smoked per day multiplied by the number of years the person has smoked. ‘Smoking time’ was defined as the total time of daily smoking. ‘Current smoker’ was defined as a negative answer to the question ‘If you stopped smoking, specify at which age you stopped’. ‘Former smoker’ was defined as a positive answer to the former question. ‘Ever used snus daily’ was defined as a positive answer to ‘Have you ever used snus on a daily basis for at least a year?’. ‘Snus use time’ was defined as a total number of years of daily snus use. ‘Current snus user’ was defined as a negative answer to the question ‘If you stopped using snus, specify at which age you stopped’. ‘Former snus user’ was defined as a positive answer to the same question. ‘Smokers’ and ‘snus users’ had to state the year when they started to use tobacco, and ‘former smokers’ and ‘former snus users’ had to state the year when they stopped using tobacco. Lag time or restrictions were not considered. ‘Asthma’ was defined by an affirmative answer to ‘Have you been diagnosed as having asthma by a physician?’ and ‘chronic obstructive pulmonary disease’ (COPD) was defined by ‘Have you been diagnosed as having Chronic obstructive pulmonary disease, COPD, by a physician?’. ‘Use of cortisone’ was defined as ever having used oral cortisone medication for at least 2 weeks. An affirmative answer to the question ‘Have you been diagnosed as having diabetes by a physician?’ defined diabetes. ‘Eye trauma’ was defined by an affirmative answer to the question ‘Have you ever had a serious eye injury, e.g., a heavy hit, sharp object, or corrosive substance?’. The year of diagnosis of asthma, COPD, diabetes, and eye trauma was required to be reported. Having a close relative who had cataract surgery was derived from ‘Do you have any close relative (biological father, mother, or sibling) that had cataract surgery?’. Myopia (nearsightedness) was defined as ‘Are you nearsighted, i.e., you can see near objects clearly, but far objects are blurry?’. The body mass index (BMI) was calculated from reported weight and height in the questionnaire using the formula BMI = mass (kg)/(height (m))^2^. Education level was derived from the answer to the question ‘What is your highest level of education?’ (low = primary school or vocational school, middle = high school or similar, high = university or similar and other = other education, folk high school or similar).

### 2.1 Statistical analysis

Demographic characteristics are presented as numbers, and percentages, for categorical variables. Continuous variables with skewed distribution, i.e. BMI, are presented as medians with IQR (Interquartile range). The prevalence of self‐reported cataract and cataract surgery was calculated. Prevalence ratios (PRs) with 95% confidence intervals (CIs) were calculated using log‐binomial regression models.

Longitudinal data analysis was enabled since information on the year of cataract surgery, ‘start’ and ‘stop’ year for smoking, use of snus, and year of diagnosis of asthma, COPD and diabetes had been obtained. The relative incidence of cataract surgery was assessed. To examine the association between smoking or snus use and incident cataract surgery, hazard ratios (HRs) with 95% CIs were estimated using a Cox regression model. Former and current smoker, and former and current snus users were included as time‐dependent variables, i.e. updated at each event time. In separate Cox regression analyses, total time of daily smoking, i.e. smoking time, pack‐years, and total time of daily snus use, i.e. snus use time, were included as continuous time‐dependent variables. Asthma, COPD, diabetes, and eye trauma were also included as time‐dependent variables, solely adjusting for variables present before the event, i.e. cataract surgery.

Log‐binomial regression and Cox regression were initially performed adjusting for smoking, snus use, education level, and gender as potential confounders (Model 1). Secondly, adjustments were made for additional potential risk factors, some of them potential confounders; asthma, COPD, corticosteroid use, diabetes mellitus, eye trauma, cataract heredity, myopia, and the BMI (Model 2). Since all participants were born in 1951, age was not adjusted for. Separate analyses were performed for men and women.

All analyses were performed using sas version 9.4M6 (SAS Institute, Cary, NC, USA).

## 3 RESULTS

Based on the 9316 individuals in the study population, an overall prevalence of self‐reported cataract (including cataract diagnosis and/or cataract surgery) was determined to 11.3% (13.5% women; 9.0% men). The prevalence of self‐reported cataract surgery was overall 6.3% (7.1% women; 5.4% men) (Table 1). Among participants, 51% were women and 49% were men. The prevalence of having ever smoked daily was 59.3%, with 61.1% in men and 57.6% in women, whilst the overall prevalence of having ever used snus daily was 20.3%, with 37.2% in men and 4.1% in women. The prevalence of being a current smoker was 12.1% and the prevalence of having formerly smoked was 47.2%. The prevalence of currently using snus was 9.8% and the prevalence of having formerly used snus was 10.5%. The prevalence of cataract in participants who had ever smoked daily was 11.9%, compared to 10.4% in those who had never smoked (Table 2). The prevalence of heredity for cataract surgery was 41.6%, myopia 30.8%, eye trauma 8.6%, asthma 7.8%, COPD 2.6%, diabetes mellitus 8.6%, and corticosteroid use 11.6% whilst the median BMI was 25.6 (IQR 23.4–28.4).

**TABLE 1:** Prevalence of self‐reported diagnosis of cataract, previous cataract surgery, ever smoking and ever snus user (gender differences).

	All, *n* = 9316	Women, *n* = 4747	Men, *n* = 4569
No cataract, % (*n*)	88.7 (8262)	86.5 (4106)	91.0 (4156)
Cataract^a^, % (*n*)	11.3 (1054)	13.5 (641)	9.0 (413)
Previous cataract surgery, % (*n*)	6.3 (584)	7.1 (335)	5.4 (249)
Ever daily smoker, % (*n*)	59.3 (5521)	57.6 (2733)	61.1 (2788)
Ever daily snus user, % (*n*)	20.3 (1890)	4.1 (193)	37.2 (1697)

*Note*: 9316 participants in Western Sweden answered a questionnaire, ‘Cataract and working environment’, in 2014–2015 about cataract diagnosis, cataract surgery, smoking, snus, eye diseases, and other diseases.

^**a**^ Self‐reported cataract diagnosis and/or cataract surgery.

**TABLE 2:** Demographics of study population.

	All, *n* = 9316	Never smoker, *n* = 3795	Ever daily smoker, *n* = 5521	Never snus user, *n* = 7426	Ever daily Snus user, *n* = 1890
Eye conditions					
Cataract, *n* (%)^a^	1054 (11.3)	396 (10.4)	658 (11.9)	866 (11.7)	188 (9.9)
Cataract surgery, *n* (%)	584 (6.3)	209 (5.5)	375 (6.8)	475 (6.4)	109 (5.8)
Heredity for cataract surgery, *n* (%)	3815 (41.6)	1620 (43.4)	2195 (40.3)	3122 (42.7)	693 (37.1)
Myopia, *n* (%)	2789 (30.8)	1260 (34.1)	1529 (28.4)	2359 (32.7)	430 (23.2)
Eye trauma, *n* (%)	801 (8.6)	251 (6.7)	550 (10.0)	541 (7.3)	260 (13.8)
Gender					
Female gender, *n* (%)	4747 (51.0)	2011 (53.1)	2736 (49.5)	4554 (61.3)	193 (10.2)
Systemic comorbidity					
Asthma, *n* (%)	724 (7.8)	273 (7.2)	451 (8.2)	561 (7.6)	163 (8.6)
COPD, *n* (%)	241 (2.6)	10 (0.3)	231 (4.2)	187 (2.5)	54 (2.9)
Diabetes mellitus, *n* (%)	801 (8.6)	271 (7.2)	530 (9.6)	584 (7.9)	217 (11.5)
Corticosteroid use, *n* (%)	1069 (11.6)	378 (10.1)	691 (12.6)	856 (11.6)	213 (11.4)
BMI					
BMI (kg/m^2^), median (IQR)	25.6 (23.4–28.4)	25.3 (23.2–28.0)	25.8 (23.6–28.6)	25.4 (23.1–28.2)	26.3 (24.4–28.7)
Education^b^					
Low education, *n* (%)	3228 (34.7)	1157 (30.5)	2071 (37.5)	2452 (33.0)	776 (41.0)
Middle education, *n* (%)	1970 (21.1)	785 (20.7)	1185 (21.4)	1544 (20.8)	426 (22.5)
High education, *n* (%)	3426 (36.8)	1585 (41.8)	1841 (33.3)	2843 (38.3)	583 (30.8)
Other education, *n* (%)	692 (7.4)	263 (6.9)	429 (7.8)	586 (7.9)	106 (5.6)

*Note*: In 2014–2015, 9316 participants in Western Sweden answered the questionnaire, ‘Cataract and working environment’, about smoking, snus, cataract, cataract surgery, eye diseases, and other diseases.

Abbreviations: BMI, body mass index; COPD, chronic obstructive pulmonary disease; IQR, interquartile range.

^a^ Self‐reported cataract diagnosis and/or cataract surgery.

^b^ Low education = primary school or vocational school, middle education = high school or similar, high education = university or similar, other education = folk high school or similar.

Log‐binomial regression analysis adjusting for gender, education, and snus use showed that participants who had ever been daily smokers had an increased prevalence of both self‐reported cataract (PR 1.16, 95% CI 1.03–1.30) and cataract surgery (PR 1.25 95% CI 1.06–1.47) compared to those who had never smoked on a daily basis (Table 3). However, having ever used snus daily was not related to cataract prevalence (adjusted for gender, education, and smoking). Similar associations were shown with log‐binomial regression adjusting for further potential risk factors; asthma, COPD, corticosteroid use, diabetes, eye trauma, heredity, myopia, and BMI; participants who had ever been daily smokers had an increased prevalence of both self‐reported cataract (PR 1.19, 95% CI 1.04–1.35) and cataract surgery (PR 1.27, 95% CI 1.06–1.53). Subgroup analyses showed that women who had ever smoked daily had an increased prevalence of cataract surgery (PR 1.30, 95% CI 1.03–1.65), and men in the same category had an increased prevalence of cataract (PR 1.27, 95% CI 1.02–1.58).

**TABLE 3:** Prevalence ratio with 95% confidence interval for self‐reported cataract diagnosis and cataract surgery.

	Cataract			Cataract surgery		
	All, PR (95% CI)	Women, PR (95% CI)	Men, PR (95% CI)	All, PR (95% CI)	Women, PR (95% CI)	Men, PR (95% CI)
Model 1						
Ever daily smoker	**1.16 (1.03–1.30)**	1.10 (0.95–1.28)	**1.25 (1.02–1.52)**	**1.25 (1.06–1.47)**	1.22 (0.99–1.52)	1.27 (0.98–1.66)
Ever daily snus user	1.03 (0.87–1.22)	1.23 (0.89–1.70)	0.96 (0.79–1.17)	0.98 (0.78–1.23)	1.33 (0.85–2.08)	0.91 (0.70–1.18)
Model 2						
Ever daily smoker	**1.19 (1.04–1.35)**	1.13 (0.97–1.33)	**1.27 (1.02–1.58)**	**1.27 (1.06–1.53)**	**1.30 (1.03–1.65)**	1.22 (0.91–1.61)
Ever daily snus user	1.02 (0.86–1.22)	1.19 (0.85–1.68)	0.98 (0.80–1.21)	0.95 (0.75–1.22)	1.25 (0.77–2.02)	0.91 (0.69–1.20)

*Note*: Logistic regression models showing the prevalence ratios and 95% CI for cataract diagnosis and cataract surgery in relation to smoking and snus use. In Model 1, adjustments for smoking, snus use, gender, and education level were performed. In Model 2, further adjustments were performed for asthma, COPD, corticosteroid use, diabetes mellitus, eye trauma, heredity for cataract surgery, myopia, and BMI. Number in bold indicates statistical significance.

Abbreviations: BMI, body mass index (kg/m^2^); CI, confidence interval; COPD, chronic obstructive pulmonary disease; PR, prevalence ratio.

The association between smoking, snus use, and the incidence of cataract surgery was assessed. Cox regression analysis adjusting for gender, education, and snus use (Model 1) showed an increased risk for cataract surgery in participants who were current smokers (HR 1.38, 95% CI 1.09–1.75). There was no significant association between having formerly smoked and cataract surgery when adjusting for gender, education, and snus use (HR 1.17, 95% CI 0.96–1.43) (Table 4). When adjusting for additional potential risk factors, there was an increased risk of cataract surgery in participants who were current smokers (Model 2) (HR 1.34, 95% CI 1.04–1.74) and those who were former smokers (HR 1.27, 95% CI 1.03–1.56). Cox regression analysis showed no significant association between current or former use of snus and cataract surgery in the whole population. Separate analyses for men and women revealed an increased risk for cataract surgery among women who were current smokers (HR 1.41, 95% CI 1.01–1.99), and among women who were current snus users, a group consisting of 108 participants (HR 2.04, 95% CI 1.16–3.60).

**TABLE 4:** Hazard Ratio based on Cox regression models for incident cataract surgery in current and former smokers, and in current and former snus users.

	All HR (95% CI)	Women HR (95% CI)	Men HR (95% CI)
Model 1			
Current smoker	**1.38 (1.09–1.75)**	**1.36 (1.00–1.85)**	1.37 (0.95–1.99)
Former smoker	1.17 (0.96–1.43)	1.15 (0.89–1.49)	1.19 (0.88–1.63)
Current snus user	0.99 (0.72–1.35)	**2.13 (1.24–3.67)**	0.79 (0.55–1.15)
Former snus user	0.99 (0.71–1.40)	0.78 (0.25–2.44)	0.98 (0.68–1.41)
Model 2			
Current smoker	**1.34 (1.04–1.74)**	**1.41 (1.01–1.99)**	1.20 (0.81–1.80)
Former smoker	**1.27 (1.03–1.56)**	1.30 (0.98–1.71)	1.24 (0.90–1.70)
Current snus user	0.96 (0.69–1.33)	**2.04 (1.16–3.60)**	0.79 (0.53–1.16)
Former snus user	0.98 (0.69–1.39)	0.82 (0.26–2.59)	0.99 (0.68–1.44)

*Note*: In Model 1, adjustments for smoking, snus use, gender, and education level were performed. In Model 2, further adjustments were performed for asthma, COPD, corticosteroid use, diabetes mellitus, eye trauma, heredity, myopia, and BMI. Number in bold indicates statistical significance.

Abbreviations: BMI, body mass index (kg/m^2^); CI, confidence interval; COPD, chronic obstructive pulmonary disease; HR, Hazard ratio.

Cox regression analysis with time‐dependent variables showed an increased risk for cataract surgery as overall time as a smoker increased. The hazard ratio for every 5 years of smoking was 1.05 (95% CI 1.02–1.08), adjusting for gender, education, and snus use (Table 5). A similar increased risk for cataract surgery was shown when adjusting for further potential risk factors (HR 1.05, 95% CI 1.02–1.08 for 5 years of smoking). Similar HR:s were seen with an increase in pack‐years, when adjusting for gender, education, and snus use (HR 1.05, 95% CI 1.01–1.08, for every 5 pack‐years), and when adjusting for additional potential risk factors (HR 1.04, 95% CI 1.00–1.08 for every 5 pack‐years). Separate analyses for men and women revealed a significant association between smoke time and cataract surgery in women (HR 1.07, 95% 1.03–1.10 for every 5 years of smoking). Cox regression analysis showed no significant association between overall snus use time and cataract surgery (HR 0.95, 95% CI 0.90–1.00 for 5 years of snus use).

**TABLE 5:** Hazard Ratio based on Cox regression models for incident cataract surgery in smokers (5 years of smoking) and snus users (5 years of snus use).

	All HR (95% CI)	Women HR (95% CI)	Men HR (95% CI)
Model 1			
Smoking time^a^	**1.05 (1.02–1.08)**	**1.06 (1.02–1.09)**	1.04 (1.00–1.08)
Snus use time^b^	0.96 (0.91–1.01)	0.97 (0.78–1.20)	0.96 (0.91–1.01)
Model 2			
Smoking time^a^	**1.05 (1.02–1.08)**	**1.07 (1.03–1.10)**	1.03 (0.99–1.08)
Snus use time^b^	0.95 (0.90–1.00)	0.99 (0.79–1.23)	0.95 (0.90–1.01)

*Note*: In Model 1, adjustments for smoking, snus use, gender, and education level were performed. In Model 2, further adjustments were performed for asthma, COPD, corticosteroid use, diabetes mellitus, eye trauma, heredity, myopia, and BMI. Number in bold indicates statistical significance.

Abbreviations: BMI, body mass index (kg/m^2^); CI, confidence interval; COPD, chronic obstructive pulmonary disease; HR, Hazard ratio.

^a^ Total time of daily smoking.

^b^ Total time of daily snus use.

## 4 DISCUSSION

In this general population cohort study of 63–64‐year‐olds in Western Sweden, smoking was associated with a dose‐dependent increased risk of cataract surgery, even after adjustment for several other potential confounders. However, there was no association between snus use and cataract except in a small subgroup of women who were current snus users.

In Sweden, snus has been commonly used since the 19th century (Nordgren & Ramström, 1990). Even though it is growing more popular among women, it has traditionally been used mostly by men (Folkhälsomyndigheten, 2021). In our study, the prevalence of men who had ever used snus was higher, 37.2%, than in women, 4.1%. The overall prevalence of having ever smoked was 59.3% (61.1% in men and 57.6% in women), which is comparable to the prevalence of 62.2% (63.3% in men and 61.2% in women) in the H70 study (Rydberg Sterner et al., 2019), a study with 70 years old participants in Gothenburg, Western Sweden. Several cross‐sectional studies show an association between cigarette smoking and the prevalence of cataract, especially nuclear cataract (Cumming & Mitchell, 1997; Hirvela et al., 1995; Klein et al., 1993). A dose–response effect between frequency and duration of smoking and the development of cataract has been observed (Hiller et al., 1997), and in our study, the hazard ratio for cataract surgery increased with 5% for every 5 years of smoking and 4% for every 5 pack‐years. It has been shown that ex‐smokers have an increased risk for cataract compared to people who have never smoked, with a decreased risk when compared to participants who are present smokers (Delcourt et al., 2000; Weintraub et al., 2002), and it has been observed that smokers who stopped smoking >10 years ago had a lower risk for cataract compared to smokers who stopped smoking <10 years ago (West et al., 1989). In our study there was no significant difference between ex‐smokers and current smokers.

To our knowledge, this is the first study to investigate if there is an association between snus use and cataract. In the present study, there was no apparent association between snus use and cataract. An increased risk for cataract surgery was observed in a small subgroup of women who were current snus users, but not in the whole group, nor was there any increased risk in men when analysing years of snus use and cataract surgery. Since the sample size of women currently using snus was small, the results should be interpreted with caution, as there may be other factors that are not fully adjusted for that could explain the association.

Smokers and snus users are both exposed to high levels of nicotine and tobacco‐specific nitrosamines (Norweigan Institute of Public Health, 2019). Since no evident effect of snus use on the development of cataract was observed, it can be hypothesised that nicotine is probably not the cause of cataract development, nor nitrosamines. On the other hand, cadmium levels are elevated in smokers, but not in consumers of smokeless tobacco in the U.S. (Marano et al., 2012; Prasad et al., 2016). Lenses with cataract have a higher concentration of cadmium compared to lenses without cataract, and it has therefore been hypothesised that cadmium can accelerate the development of cataract via cadmium accumulation in the lens, causing direct toxicity (Cekic, 1998).

The overall prevalence of cataract and cataract surgery in this study was lower compared to the Swedish H70 study (Nordström et al., 2022), where the overall prevalence of cataract was 23.4%, 27.2% in women and 19.1% in men. The lower prevalence of cataract in our study can probably be attributed to the overall lower age of participants as compared to the H70‐study participants. Cataract increases with age, and the average age for cataract surgery in Sweden in 2022 was 74.6 years (Behndig et al., 2022). With time, lens proteins aggregate and oxidative damage occur which leads to age‐related cataract (Bloemendal et al., 2004). With increasing age, the proliferation of outer lens layers leads to stiffening and compression of the central nucleus, i.e. nuclear sclerosis, which is the most common type of cataract. The prevalence of cataract surgery was slightly lower in our study compared to a prevalence of 8% in 63–64 year olds in the year of 2022 (Bro et al., 2024), which could be due to the increase in cataract surgeries in Sweden in recent years. The prevalence of cataract and cataract surgery in our study was slightly higher in women than in men, which is in accordance with the study on 70‐year‐olds in Gothenburg (Nordström et al., 2022), and the Swedish National Cataract register that shows more women than men have cataract surgery (Behndig et al., 2022). An increased risk for cataract surgery in women could be due to a withdrawal effect of oestrogen in menopause (Zetterberg & Celojevic, 2015).

The main strengths of our study include a large cohort size, a population‐based design, and a relatively high response rate. All individuals in the Västra Götaland County born in 1951 were invited to participate in the study. The ratio between men and women was in accordance with the total same‐age population in the Västra Götaland County according to Statistics Sweden, SCB; 49% men and 51% women (SCB, 2014). The proportion of participants who had ever smoked was similar to that found in the H70 study from Western Sweden where participants were born in 1944. The proportion of participants with higher education (university or similar) was somewhat higher, 37%, than that of the total same‐age population in the Västra Götaland County, 32% (SCB, 2014). In summary, we think the present study's representativeness is relatively good and that the results could likely be generalised to other similarly aged populations in Sweden. Another strength of the study was the possibility to adjust for several potential confounders, which adds robustness to the results. The fact that we also studied the incidence of cataract surgery adds longitudinal data value both when it comes to the outcome and most included covariates.

There were also potential limitations in our study. No global standard exists for the definition of cataract, and no information on cataract subtypes was available in this study. Self‐reported information has a potential risk for bias. However, an earlier study shows a high validity for self‐reported cataract surgery, including a higher sensitivity and specificity for self‐reported cataract surgery, 90.7% and 99.2%, than for self‐reported cataract diagnosis, 52.9% and 92.3% (Nordström et al., 2022). Hence, self‐reported cataract surgery was used as an outcome. Self‐reported cataract surgery ought to be more clinically relevant than self‐reported cataract, as it is more likely that people remember the year of cataract surgery rather than which year they were told that had a cataract by an ophthalmologist or optician. Since the subgroup of women who were current snus users is of limited sample size, these findings should be interpreted with caution, but further studies with a larger sample size may provide clarification. Similarly, the statistical analyses do not include participants who smoked or used snus occasionally, and hence this study does not include data on any possible association between cataract and occasional smoking and snus use. Nor do the analyses include a measure of snus amount used, other than in time (years).

In summary, this study confirms findings from previous studies that smoking is associated with an increased prevalence of cataract and cataract surgery, suggesting a dose‐dependent increased risk of incident cataract surgery, adjusting for several potential confounders. As the first to report, to our knowledge, no clear association between snus use and cataract was found.

## FUNDING INFORMATION

This work was supported by grants from the Swedish state under the agreement between the Swedish government and the county councils, the ALF‐agreement (ALF‐GBG‐725041 and 966230), The Gothenburg Society of Medicine, Dr Reinhard Marcuses Foundation, Konung Gustaf V:s och Drottning Victorias Frimurarestiftelse, De Blindas Vänner, Agneta Prytz‐Folkes och Gösta Folkes stiftelse and Kronprinsessan Margaretas Arbetsnämnd för Synskadade. The sponsor or funding organisation had no role in the design or conduct of this research.
